# Evolving AAV-delivered therapeutics towards ultimate cures

**DOI:** 10.1007/s00109-020-02034-2

**Published:** 2021-02-16

**Authors:** Xiangjun He, Brian Anugerah Urip, Zhenjie Zhang, Chun Christopher Ngan, Bo Feng

**Affiliations:** 1 School of Biomedical Sciences, Faculty of Medicine; Institute for Tissue Engineering and Regenerative Medicine (iTERM), The Chinese University of Hong Kong, Shatin N.T., Hong Kong SAR, China; 2 Centre for Regenerative Medicine and Health, Hong Kong Institute of Science & Innovation, Chinese Academy of Sciences, Shatin N.T., Hong Kong SAR, China; 3 Guangzhou Regenerative Medicine and Health Guangdong Laboratory, Guangzhou, 510320 China; 4 Guangzhou Institute of Biomedicine and Health, Chinese Academy of Sciences, Guangzhou, 510530 China

**Keywords:** Gene therapy, Adeno-associated virus, Gene transfer, Gene editing

## Abstract

Gene therapy has entered a new era after decades-long efforts, where the recombinant adeno-associated virus (AAV) has stood out as the most potent vector for in vivo gene transfer and demonstrated excellent efficacy and safety profiles in numerous preclinical and clinical studies. Since the first AAV-derived therapeutics Glybera was approved by the European Medicines Agency (EMA) in 2012, there is an increasing number of AAV-based gene augmentation therapies that have been developed and tested for treating incurable genetic diseases. In the subsequent years, the United States Food and Drug Administration (FDA) approved two additional AAV gene therapy products, Luxturna and Zolgensma, to be launched into the market. Recent breakthroughs in genome editing tools and the combined use with AAV vectors have introduced new therapeutic modalities using somatic gene editing strategies. The promising outcomes from preclinical studies have prompted the continuous evolution of AAV-delivered therapeutics and broadened the scope of treatment options for untreatable diseases. Here, we describe the clinical updates of AAV gene therapies and the latest development using AAV to deliver the CRISPR components as gene editing therapeutics. We also discuss the major challenges and safety concerns associated with AAV delivery and CRISPR therapeutics, and highlight the recent achievement and toxicity issues reported from clinical applications.

## Development of AAV vector for gene therapy

Adeno-associated virus (AAV) is a small, non-enveloped, single-stranded DNA virus that belongs to the genus Dependovirus in the Parvovirus family. AAV was initially discovered in 1965 as a contaminant of an adenovirus (AdV) preparation [1], and later identified as a new infectant after antibodies to different AAV serotypes were detected in children [2]. As a dependovirus, AAV is replication-defective in the absence of a helper virus such as adenovirus or herpes virus. During the latent phase, AAV will integrate into the host cell genome and remain dormant until co-infection occurs and triggers viral replication [3]. The wild-type AAV (wtAAV) contains a single-stranded DNA genome approximately 4.7 kb in length, which consists of *rep* and *cap* genes flanked by two inverted terminal repeats (ITRs). In later years, studies found that the AAV genes could be expressed without integration, and a recombinant DNA sequence between the AAV2 ITRs in an engineered vector could be successfully encapsulated into pseudovirus by providing *rep* and *cap* genes *in trans* [3]. As a result, the engineered AAV vectors possess the capacity to carry a recombinant genome up to ~ 4.7 kb, and can be pseudotyped with ease using different *cap* genes to generate virions with desired tissue tropisms. Similar to wtAAVs, the recombinant AAVs are low-immunogenic and non-pathogenic, while due to the lack of other viral elements, the AAV vector genome largely remains non-integrative in host cells. Collectively, these features make the AAV system an ideal delivery tool for in vivo gene transfer and gene augmentation therapy [4].

The first AAV-based in vivo gene delivery was reported in 1993 when Flotte et al. stably expressed cystic fibrosis membrane conductance regulator (CFTR) in rabbit lung for up to 6 months [5]. The promising results led to the first phase I clinical trial of AAV-based gene therapy in 1996 which delivered the *CFTR* gene for treating patients with cystic fibrosis [6]. In 2000, another early study reported the successful delivery of human factor IX (hFIX) using AAV vectors to ameliorate the bleeding symptoms in patients with hemophilia B [7], although the follow-up study of these patients revealed elevated levels of neutralizing antibodies (NAbs) against AAV vectors, which abolished the long-term efficacy of the treatment [8]. As a result, subsequent studies focused on exploring various strategies to overcome the issues associated with adaptive immune responses to AAV vectors. Eventually, successful therapeutic gene transfer was achieved by selectively recruiting patients with low NAbs and administrating short-term anti-T cell immunosuppressive agents [9, 10].

## Recent progress of AAV-based gene therapy in clinics

**Fig. 1:**
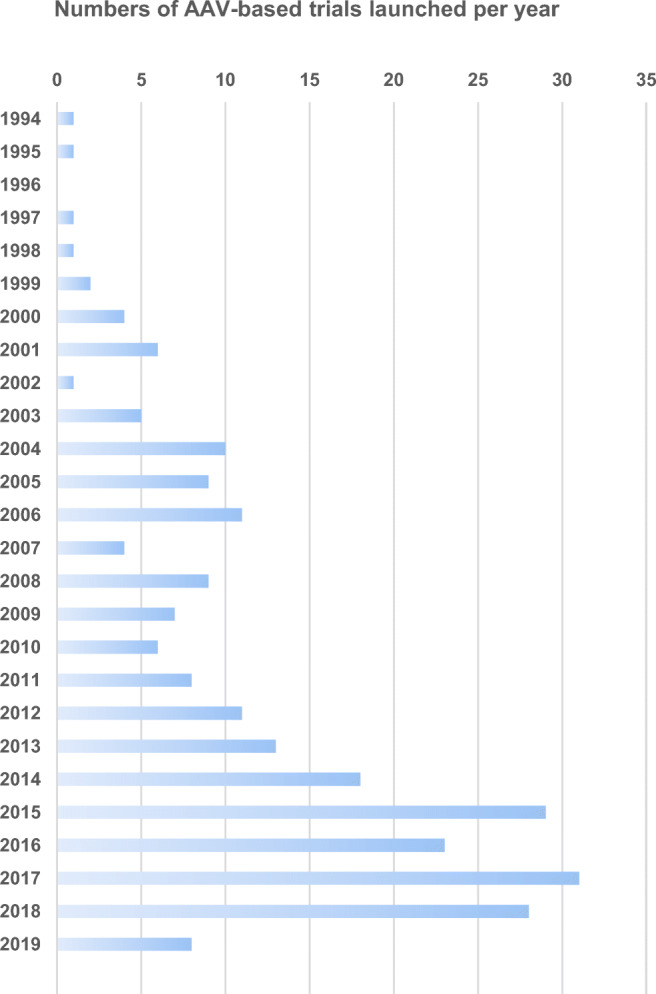
Numbers of clinical trials using AAV vectors for gene transfer. *Data were obtained from http://www.abedia.com/wiley/index.html

Over the last decade, AAV gene therapy has accomplished remarkable progress and is finding its way into medical practice (Fig. 1). In 2012, the European Medicines Agency (EMA) approved the first-ever AAV-based gene therapy Glybera [11], a recombinant AAV product that delivers the human lipoprotein lipase (*LPL*) gene to treat hereditary lipoprotein lipase deficiency (LPLD) [12]. Five years later, Luxturna (voretigene neparvovec-rzyl), another AAV gene therapy for *RPE65*-mediated inherited retinal dystrophy, was approved by the United States Food and Drug Administration (FDA) and entered the US market [13, 14]. Recently, Zolgensma (onasemnogene abeparvovec), an AAV product delivering a functional copy of the *SMN1* gene for spinal muscular atrophy type 1 (SMA1), was approved by the FDA in 2019 and became the third AAV-based gene therapy product in the market [15–17] (Table 1).

**Table 1:** Approved AAV gene therapy products

Product name	Year of approval, agency	Manufacturer	Gene to deliver	Disorder/condition	AAV vector	Clinical trials	Clinical outcome	Ref
Glybera™ (alipogene tiparvovec)	Nov 2012, EMA	uniQure	LPL	Lipoprotein lipase deficiency (LPLD)	ssAAV1	CT-AMT-010-01 CT-AMT-011-01 CT-AMT-011-02	Postprandial total plasma triglyceride (TG) levels were reduced by 27% and 41% in patients at the dose of 1 × 10^11^ and 3 × 10^11^ vg/kg respectively. In subjects receiving a dose of 1 × 10^12^ vg/kg, a more significant reduction in TG levels, of 60% (from 18.77 to 7.30 mM), was confirmed	[12]
Luxturna™ (voretigene neparvovec-rzyl)	Dec 2017, FDA	Spark Therapeutics	RPE65	Hereditary retinal dystrophy	ssAAV2	NCT00516477 NCT01208389 NCT00999609	Significant vision improvement was reported in all patients at 1 year after the 40 subjects received 1.5 × 10^11^ vg per eye in at least one eye via subretinal injection	[13, 14]
Zolgensma™ (onasemnogene abeparvovec-xioi)	May 2019, FDA	AveXis	SMN1	Spinal muscular atrophy type 1 (SMA1)	scAAV9	NCT02122952 NCT03306277 NCT03421977 NCT03505099	Seven of twelve infants who received the treatment were free of permanent ventilation 2 years later. Swallow function in 11 infants was stable or improved. Eleven patients achieved full head control and unassisted sitting, and two patients could walk independently. The mean proportion of time hospitalized and unadjusted annualized hospitalization rate were reduced significantly (4.4%, 2.1)	[15–17]

The continuous development of AAV vectors has provided an excellent treatment modality for inherited ocular disorders. The eye is considered an attractive target organ due to (i) high accessibility for AAV delivery; (ii) immune-privileged environment maintained by the blood-retinal barrier; and (iii) enclosed structure and small tissue size which allows using lower vector dose to achieve therapeutic benefits [18]. As a result, the number of new clinical trials for ocular gene therapies has been steadily increasing for the past 5 years. Among the ongoing trials, approximately 80% are currently in phase 1/2, with estimated 70% (more than 40 trials) that are using AAV vectors (https://clinicaltrials.gov/) [18].

Gene therapy trials for hemophilia have been actively pursued since the 1990s. The earlier trials showed that AAV delivery was well tolerated in human patients with transient elevation of liver enzymes which then returned to the normal level without manifesting adverse side effects [7]. The clinical data also revealed that the presence of NAbs against natural AAVs abolished the efficacy of the AAV vector-delivered treatment [8]. These findings suggested to adopt strategies such as selective recruitment of patients with low NAbs and co-administration with a short-term anti-T cell suppressants in subsequent clinical trials, which eventually led to the first long-term success for hemophilia B gene therapy in 2011 [9, 10]. The study recruited patients with severe hemophilia B and divided them into three groups to receive a single dose of scAAV2/8-LP1-hFIXco vectors at 2 × 10^11^, 6 × 10^11^, and 2 × 10^12^ vg/kg. The patients from all groups exhibited a stable dose-dependent increase in FIX levels [10]. Markedly, in the high-dose cohort, all six patients showed a consistent increase of plasma FIX that reached 5.1 ± 1.7% of normal level together with bleeding episodes reduced by more than 90% [10]. Seven years later, a follow-up investigation confirmed stable maintenance of the FIX levels and hemostasis in all of these patients [19]. These promising results prompted subsequent clinical trial programs to optimize the AAV delivery and input dose. UniQure carried out a clinical trial delivering the scAAV2/8-LP1-hFIXco vector using AAV5 capsid (AMT-060) at the dose of 5 × 10^12^ and 2 × 10^13^ vg/kg, and achieved therapeutic efficacy and safety profile similar to the previous clinical trial using the AAV8 capsid (NCT02396342; EudraCT2013-005579-42) [20]. Spark Therapeutics used a modified-AAV8 capsid (SPK-9001) to deliver hyperactive FIX variant (R338L) Padua in a clinical trial for hemophilia B, which further reduced the input dose (5 × 10^11^ vg/kg) to achieve sustained FIX activity at around 33.7 ± 18.5% of the normal level [21]. These results provided the data necessary for expanding the clinical trial for SPK-9001 into phase 3 (NCT03587116) as well as testing AAV5- and AAVS3-delivered hFIX-Pauda (NCT03489291, NCT03369444) in new phase 1 trials, where long-term monitoring will be necessary to determine the efficacy of the treatment [22].

The advancement of AAV gene therapy for hemophilia B has also expedited the approvals of clinical trials for hemophilia A. In 2017, BioMarin Pharmaceutical reported the success of their first phase 1/2 clinical trial using AAV5 to deliver the codon-optimized B domain-deleted F8 cDNA (BDD-FVIII) (BMN 270) into patients with severe hemophilia A and achieved FVIII activity at around 77% of the normal level in the high-dose (6 × 10^13^ vg/kg)-treated group [23]. Immediately, BioMarin expanded the testing of BMN270 into two phase 3 trials, with an estimated 170 patients enrolled for the studies (NCT03370913, NCT03392974). In the following year, Spark Therapeutics quickly followed and unveiled the outcome of their phase 1/2 clinical trial using an engineered capsid AAV-LK03 to deliver BDD-FVIII (SPK-8011) with five out of seven patients treated with the high-dose vectors (2 × 10^12^ vg/kg) exhibited FVIII at 16–49% of the normal levels [24].

The research using hemophilia as a study model has pioneered liver-based gene therapy [22, 25]. The promising results from optimizing different aspects of recombinant AAV for gene delivery, including vectors, capsids, route, dose, toxicity, and immunogenicity, have paved the way for developing gene therapy for many other liver-based inherited diseases which have led to a number of clinical trials to date [26]. These include the AAV-based treatments for alpha 1-antitrypsin (AAT) deficiency, phenylketonuria (PKU), ornithine transcarbamylase deficiency, Crigler-Najjar syndrome, homozygous familial hypercholesterolemia (FH), and glycogen storage disease type Ia (GSD1a) (https://clinicaltrials.gov/) [27, 28].

A number of trials have also been undertaken to explore the therapeutic potential of AAV-based gene delivery into the central nervous system (CNS) to treat various neurological disorders. In the past, AAV delivery targeting the CNS had been challenging due to the protection of the blood-brain barrier (BBB). Since 2009, a series of studies have addressed the issue of BBB by delivering the AAV9 vectors through the intracerebroventricular or intravenous route, which yielded significant GFP expression in mouse CNS and achieved therapeutic expression of SMN, a gene responsible for the inherited neuromuscular disorder SMA1 [29, 30]. Notably, clinical testing on the AAV9-delivered SMN expression for treating SMA1 [31] has led to the remarkable success of Zolgensma, the AAV gene therapy product approved by the FDA in Dec 2019 [17]. Currently, AAV vectors have been tested in clinical trials for a number of neurological disorders**.** AAV-based gene therapy using AAV9 and AAVrh10 vectors for Sanfilippo syndrome type A (MPSIIIA) had passed safety tests and entered phase 2/3 clinical trials (NCT04360265; NCT03612869) [32]. Additionally, AAV2-delivered aromatic l-amino acid decarboxylase (AADC) gene therapy has completed phase 1/2 trial for pediatric patients with AADC deficiency (NCT02852213, NCT02926066) [33]. The safety profile and therapeutic potential of AAV-based gene therapy in monogenic neurological disorders have prompted researchers to expand gene therapy clinical trials to treat more complex neurological diseases, such as Parkinson’s disease and Alzheimer’s disease (https://clinicaltrials.gov/) [34].

It is noteworthy that hemophilia is low-hanging fruit because a very low level of gene transfer and expression is sufficient to confer therapeutic benefits [22, 25]. In many other diseases, much higher AAV doses are needed to convey therapeutic benefits from transgenes expression and have been reported to be associated with severe toxicity [15, 16, 26]. In a recent study, a clinical trial named “AT132” injected AAV8 at 1 × 10^14^ and 3 × 10^14^ vg/kg to treat X-linked myotubular myopathy (NCT03199469). Six patients treated with low-dose AAV8 showed significant improvement in motor functions. However, three boys from the high-dose group all died from progressive liver dysfunction followed by sepsis [35]. These clinical deaths highlight the risks associated with intravenous administration of high-dose AAVs and prompt a more thorough assessment for patient recruitment, such as taking account of genetic pre-deposition and pre-existing liver disease [36] (Table 2). Together, further investigations are warranted to reduce input dose; the strategies include but are not limited to (i) enhancing transgene expression, (ii) improving viral capsid and cell-type-specific promoters, and (iii) administrating immunosuppressive agents to eliminate NAbs.

**Table 2:** AAV dose and serotypes used for systemic treatment in clinical trials

Gene therapy names	Disease	Gene to be delivered	AAV serotype	AAV dose (vg/kg)	Age and delivery route	Remarks	Clinical trials	Year	Sponsor/manufacture	Ref
Hemophilia										
scAAV2/8-LP1-hFIXco	Hemophilia B	hFIXco	AAV8	2 × 10^11^, 6 × 10^11^, 2 × 10^12^	≥ 18 years, males; i.v. infusion	Confirmed efficacy and safety	NCT00979238; phase 1	2009	UCL, St Jude Children’s Res Hospital; Children’s GMP in Memphis	[9, 10, 19]
FLT180a	Hemophilia B	hFIXco	AAVS3	4.5 × 10^11^ 7.5 × 10^11^ 9.75 × 10^11^ 1.5 × 0^12^	≥ 18 years, males; i.v. infusion	Confirmed efficacy and safety	NCT03369444; phase 1	2017	UCL	[22]
AMT-060	Hemophilia B	hFIXco	AAV5	5 × 10^12^, 2 × 10^13^	≥ 18 years, males; i.v. infusion	Confirmed efficacy and safety	NCT02396342; EudraCT2013-005579-42; phase 1/2	2015	UniQure	[20]
AMT-061	Hemophilia B	hFIXco-Padua	AAV5	2 × 10^13^	≥ 18 years, males; i.v. infusion	Confirmed efficacy and safety	NCT03489291; phase 2 NCT03569891; phase 3	2018	UniQure	[22, 37]
SPK-9001	Hemophilia B	hFIXco-Padua	AAV8	5 × 10^11^	≥ 18 years, males; i.v. infusion	Confirmed efficacy and safety	NCT02484092; phase 1/2	2015	Spark Therapeutics and Pfizer	[21]
AAV-Spark100; SB-525	Hemophilia B; Hemophilia A	hFIXco-Padua; BDD-FVIII	AAV6	n.a.	18–64 years, males; i.v. infusion	n.a.	NCT03587116; phase 3	2018	Pfizer	n.a.
BMN270 (AAV5-hFVIII-SQ)	Hemophilia A	BDD-FVIII	AAV5	6 × 10^12^, 2 × 10^13^, 4 × 10^13^, 6 × 10^13^,	≥ 18 years, males; i.v. infusion	Confirmed efficacy and safety	NCT02576795, EudraCT2014-003880-38 Phase 1/2	2015;	BioMarin	[23]
BMN 270-301	Hemophilia A	BDD-FVIII	AAV5	6 × 10^13^	≥ 18 years, males; i.v. infusion	Confirmed efficacy and safety	NCT03370913, phase 3	2017	BioMarin	[38]
BMN270-302	Hemophilia A	BDD-FVIII	AAV5	4 × 10^13^	≥ 18 years, males; i.v. infusion	n.a.	NCT03392974; phase 3	2018	BioMarin	n.a
SPK-8011	Hemophilia A	BDD-FVIII	AAV-LK03	5 × 10^11^, 1 × 10^12^, 2 × 10^12^	≥ 18 years, males; i.v. infusion	Confirmed efficacy and safety	NCT03003533; phase 1/2	2016	Spark Therapeutics	[24]
PF-07055480	Hemophilia A	BDD-FVIII	AAV6	3 × 10^13^	18–64 years, males; i.v. infusion	Confirmed efficacy and safety	NCT04370054; phase 3	2020	Pfizer	[39]
Spinal muscular atrophy (SMA)										
Zolgensma (AVXS-101)	SMA	SMN	AAV9	6.7 × 10^13^; 2 × 10^14^	≤ 6 months i.v. infusion	Confirmed efficacy and safety FDA approved	NCT02122952; phase 1	2014	AveXis,	[15]
Zolgensma (AVXS-101)	SMA	SMN	AAV9	n.a.	≤ 6 months i.v. infusion	Confirmed efficacy and safety FDA approved	NCT03306277; phase 3 NCT03461289; phase 3	2017; 2018	AveXis,	[40, 41]
Zolgensma (AVXS-101)	SMA	SMN	AAV9	n.a.	≤ 6 months i.v. infusion	n.a.	NCT03837184; phase 3	2019	Novartis	n.a
Zolgensma (AVXS-101)	SMA	SMN	AAV9	1.1 × 10^14^	≤ 42 days i.v. infusion	Confirmed efficacy and safety FDA approved	NCT03505099; phase 3	2018	Novartis	[42]
Systemic treatment for other inherited diseases										
BMN 307	PKU	PAH	n.a.	3 dose levels not disclosed	≥ 15 years, i.v. infusion	n.a.	NCT04480567; phase 1/2	2020	BioMarin	n.a
HMI-102	PKU	PAH	AAVHSC15	3 dose levels not disclosed	18–55 years, i.v. infusion	n.a.	NCT03952156; phase 1/2	2019	Homology Medicines,	n.a
DTX301 (scAAV8OTC)	OTC deficiency	OTC	AAV8	2 × 10^12^; 6 × 10^12^; 1 × 10^13^	≥ 18 years, i.v. infusion	n.a.	NCT02991144; phase 1/2	2016	Ultragenyx	n.a
GNT0003	Crigler-Najjar syndrome	UGT1A1	AAV8	2 dose levels not disclosed	≥ 9 years, i.v. infusion	n.a.	NCT03466463; phase 1/2	2018	Genethon	n.a
AAV-hLDLR	Familial hypercholesterolemia	LDLR	AAV8	n.a.	≥ 18 years, i.v. infusion	n.a.	NCT02651675; phase 1/2	2016	Regenxbio	n.a
DTX401	GSD1a	G6PC	AAV8	3 dose levels not disclosed	≥ 18 years, i.v. infusion	n.a.	NCT03517085; phase 1/2	2018	Ultragenyx	[43]
ABO-102 (scAAV9.U1a.hSGSH)	MPS IIIA	SGSH	AAV9	0.5 × 10^13^ 1 × 10^13^ 3 × 10^13^	≥ 6 months, i.v. infusion	Signs of efficacy	NCT02716246; phase 1/2 NCT04360265 (follow-up)	2016; 2020	Abeona Therapeutics	[44]
AT132	X-linked myotubular myopathy	hMTM1	AAV8	1 × 10^14^ 3 × 10^14^	≤ 5 years, males; i.v. infusion	Confirmed efficacy and safety for the low-dose group; three deaths from the high-dose group	NCT03199469; phase 1/2	2017	Audentes Therapeutics	[35]

## The current limitations and future of AAV-based gene therapy

### Rapid loss of episomal AAV vectors in proliferating cells

The recombinant AAV vectors do not integrate into the host DNA but mainly remain as episomes in the transduced cells to stably express transgene for a prolonged period in somatic tissues [45]. However, among the tissues that are at either a growing stage or undergoing continuous turnover, cell proliferation will result in a fast and significant loss of transgene expression due to the dilution of non-replicative AAV vectors [46]. As a result, AAV-based gene delivery is rarely used to target fast-proliferating cells such as hematopoietic progenitors and stem cells. Evidences from multiple clinical studies on AAV-based gene therapy in adult patients with hemophilia B have also reported expression loss, despite a significant reduction in bleeding episodes [26]. Young children are seldom recruited in clinical trials for AAV-based gene therapy due to general safety concerns as well as vector dilution [10, 21]. Currently, there is limited data on the long-term efficacy of AAV gene therapy for the younger age group, which, therefore, warrants further investigations to overcome the challenges as mentioned above.

### Small packaging capacity of AAV vectors for in vivo gene delivery

Recombinant AAVs are the leading platform for in vivo gene delivery. The most commonly used AAV vector is derived from wtAAV2 with a maximum packaging capacity of ~ 4.7 kb [4]. Several genes used in gene therapy such as dystrophin (for Duchenne muscular dystrophy), FVIII (for hemophilia A), and ABCA4 (for an inherited retinal degeneration) exceed the packaging capacity of AAV and, hence, are difficult to be packaged efficiently. To overcome the size limitation, truncated versions of the transgenes, such as the BDD-FVIII, together with mini promoters and polyA signals, were generated [23]. However, the gene truncation approach was not applicable to the mini-dystrophin gene with a size range of 6–8 kb after the removal of multiple internal regions [47]. Zhang et al. reported a dual-AAV vector approach to deliver split mini-dystrophin genes that will undergo trans-splicing to synthesize the mini-dystrophin protein inside the host cells [48], based on the rationale that the AAV genome undergoes concatemerization in host cells through homologous recombination between their ITR sequences [49]. In order to improve the transfer efficiency of larger genes, intein-mediated trans-splicing technology was also developed [50] and has shown promising gene transfer efficiency in preclinical studies [47, 51].

AAV vector engineering has also aimed at overcoming the slow onset of transgene expression attributed to the time-consuming conversion of single-stranded to double-stranded AAV genome [52]. The rate-limiting step of second-strand synthesis was resolved by introducing mutations into the ITR regions to prevent terminal resolution, thereby promoting self-complementation [53]. The generation of self-complementary AAV (scAAV) vectors allows quicker expression and greater persistence in target cells [53, 54]. The clinical application of scAAV has contributed to the aforementioned triumphant in achieving long-term therapeutic benefits in AAV gene therapy for hemophilia B [9, 10] and the FDA approval of Zolgensma [15–17].

### Host immune response against AAV

AAVs are highly prevalent, with up to 70% of the world population are positive for AAV serotype 2. Although AAVs have not been reported to cause any clinical disease, humans infected with AAVs often develop immunological memory that renders AAV-based gene therapy ineffective [55].

#### Humoral immunity against AAV

It is estimated that a considerable proportion of individuals will develop humoral immunity against wtAAV in their lifetime [56]. Among the thirteen naturally occurring AAV serotypes, approximately 70% of the world population are seropositive for AAV1 and AAV2, 45% for AAV6 and AAV9, and 38% for AAV8 [55]. The prevalence of individual serotypes varies across geographical locations [57]. Infants often carry maternal anti-AAV antibodies which decline gradually in a few months after birth [58]. In AAV-based gene therapy, the pre-existing anti-AAV antibodies will recognize and prevent the AAV capsid epitopes from interacting with receptors on the recipient cells, and thus can have a profound impact on cellular transduction and gene delivery efficiencies [59]. Although the pre-existing NAbs may not inhibit gene transfer administered via the eye or parenchyma route, intravenous gene therapy for hemophilia has shown that low titers of pre-existing anti-AAV antibodies are sufficient to neutralize the AAV vectors and abolish the treatment efficacy [60, 61]. Nowadays, prescreening is routinely conducted on clinical trial subjects before receiving AAV-based gene therapy, where subjects with high NAbs, approximately 20–50% in the tested patients as estimated based on the NAbs prelavance [55], are excluded from participating in the majority of the studies [62]. Moreover, re-administration of the same AAV vector is challenging, as previously treated patients develop NAbs which inhibit the efficacy of subsequent treatment [8, 63]. Currently, several clinical trials are testing multiple AAV capsids from different AAV serotypes to deliver the hFIX and hFIX-Padua genes [22, 25, 26] to avoid being targeted by pre-existing NAbs. Generally, NAbs are specific to individual AAV capsids [63]. However, seroprevalence analysis showed that some NAbs exhibited broad cross-reactivity [64], which could render AAV serotypes switching ineffective.

#### Cellular immunity against AAV

AAV alone does not induce significant inflammatory reactions to trigger cellular immune response. Whereas, AAV co-infected with helper viruses will activate CD4+ and CD8+ T cells which then leaves a pool of memory T cells throughout the lifetime [65]. The number of people with pre-existing memory T cell to wtAAVs is far lesser than those carrying NAbs, but the presence of these AAV-specific memory T cells contributes to the different responses to the AAV treatement as observed in human and experimental animals [62]. Unlike the NAbs that are largely specific to individual capsids, the memory T cells recognize AAV epitopes that are highly conserved across serotypes [66]. Fortunately, gene therapy studies in patients with hemophilia B demonstrated the effectiveness of using immunomodulating drugs to temporarily suppress T cell responses to the AAV capsids [9, 10], and had achieved long-term FIX expression in patients that alleviated hemophilia B-related symptoms [10, 19]. Overall, the results from these trials highlight the important role of memory T cells in influencing the treatment efficacy of AAV-based gene therapy.

## Overcoming the current hurdles in AAV-based therapy

### Capsid engineering to evade pre-existing AAV antibodies

Seroprevalence analyses indicate that some NAbs can cross-react with multiple wtAAV serotypes [67], and thus, switching to other naturally occurring AAV serotypes offers limited benefits for immune evasion. Studies on capsid biology revealed distinctive residues that are responsible for antibody binding [54], suggesting that AAV capsid engineering could potentially be the solution to overcome the immune barriers [68].

There are two main strategies for AAV capsid engineering: rational protein design and directed evolution [54]. Rational protein design relies on the prior knowledge of the capsid amino acid sequences and their functionalities. For example, a monoclonal antibody A20 was identified to bind AAV2 through residue 265 of VP1 protein. By inserting a different amino acid at residue 265, a mutant AAV2.5 was generated with a weaker binding affinity to A20 [69]. Directed evolution uses error-prone PCR or DNA shuffling strategy to construct a library of mutant capsids and perform a rigorous selection to identify desired mutants [54]. For instance, AAV-DJ is a chimera capsid generated from AAV serotypes 2, 8, and 9 through DNA shuffling , which supports gene delivery with higher efficiency than AAV2 into the liver of both naïve and IVIG (intravenous immunoglobulin) treated mice [70]. Another example of engineered immune-evading AAV is the novel variant SCH9 which can efficiently transduce neural stem cells (NSCs) and is ten times more resistant to NAbs than the parental AAV9 [71]. Collectively, the efforts to diversify AAV capsids put forth a hopeful future for creating more efficient vectors.

Besides capsid engineering, alternative approaches have also been explored to overcome the NAb issue. For instance, recent reports showed that plasmapheresis could efficiently remove NAbs to permit AAV re-delivery and transgene expression in rodent models, which can serve as an alternative solution when anti-AAV NAbs cannot be bypassed through other means [72, 73]. Nonetheless, host immune responses to intravenous AAV delivery is far more complex than previously known [67]. Hence, further research is necessary to unlock the full potential of in vivo gene therapy.

### Coupling AAV with integrative vectors to support long-term expression

In parallel with the development of AAV, other delivery vectors such as lentivirus, retrovirus, and transposon system are well-established systems and have been adopted for therapeutic applications [74–76]. These vectors have relatively large payloads and can integrate into the genome of both dividing and non-dividing cells to support long-term gene expression. In clinics, integrative vectors have been implemented in ex vivo therapies, to engineer immune cells to treat patients with end-stage cancers [77, 78] or to correct deleterious genetic defects in highly proliferative hematopoietic progenitor cells [79, 80]. However, the application of integrative vectors for in vivo gene transfer is limited.

By taking advantage of the highly efficient AAV-mediated gene delivery system, a hybrid AAV/piggyBac vector system was developed and successfully introduced stable transgene transposition into hepatocyte genome [81, 82]. The high efficiency of in vivo gene delivery using the hybrid AAV/piggyBac vector and stable transgene expression was evident in the livers of neonatal mice, which resulted in the correction of the two genes responsible for inherited urea cycle defects [81]. Recently, the AAV/piggyBac hybrid vector has been adopted to treat cystic fibrosis in pigs using aerosolized AAV vectors carrying *CFTR* flanked by the piggyBac terminal repeats [83]. Despite the small packaging capacity of AAV and semi-random integrations of transposons, the hybrid AAV/piggyBac vector system offers a stable and long-term transgene expression in treated animals through transgene integrations in transduced cells.

## Advancement of in vivo gene editing using AAV-delivered nucleases

### Breakthrough in genome editing technologies

The newly developed engineered nucleases, such as zinc-finger nucleases (ZFNs) [84], transcription activator-like effector nucleases (TALENs) [85], and type II bacterial clustered regularly interspaced short palindromic repeats (CRISPR)-associated protein 9 (Cas9) system [86], have revolutionized genome editing technologies and opened up a new avenue for the advancement of targeted gene editing therapy. Both ZFNs and TALENs contain a FokI nuclease domain and an assembly of multiple motifs that are programmable to recognize a selected DNA sequence to guide site-specific cleavage. Distinctly, the CRISPR/Cas9 system utilizes a single guide RNA (sgRNA), complexed with Cas9 nuclease, to recognize a variable 20-nucleotide target DNA sequence adjacent to a protospacer adjacent motif (PAM), and cuts the target DNA [87, 88]. The ZFN, TALEN, and CRISPR/Cas9 can all induce DNA double-strand break (DSB) efficiently at a pre-selected target site, which is then repaired via one of the two main mechanisms, the non-homologous end joining (NHEJ) or homology-directed repair (HDR) pathway [89, 90].

The NHEJ repair is error-prone and introduces small insertions or deletions (indels) at the targeted cleavage points [89], thereby abolishing the function of a target gene [91]. Distinctly, the HDR pathway relies on existing homologous DNA sequences to direct DNA repair through a strand-exchange process [90], which supports the replacement of genome segments with donor DNA based on the flanking homology sequences [92]. The site-specific gene targeting by ZFN, TALEN, and CRISPR/Cas9 systems have been widely adopted in research to introduce a wide range of genomic modifications, such as targeted mutation, insertion, and large deletion [93]. The CRISPR/Cas9 system is particularly praised by the scientific community for its superior simplicity, ease for reprogramming, and robust performance, which has garnered more popularity than other nuclease tools [94, 95].

### Therapeutic potential of using AAV-delivered nucleases for in vivo gene editing

The highly customizable and robust gene editing nucleases present appealing opportunities to develop novel therapeutics. As a proof-of-concept, the AAV-delivered ZFN system was first employed to introduce somatic gene editing in mice for disease corrections [96]. Several studies conducted by K. High et al. have provided concrete evidence demonstrating that somatic gene targeting was able to achieve transgene integration, long-term expression, as well as restoration of hemostasis in mice with hereditary hemophilia [96–98].

Subsequently, AAV-delivered CRISPR/Cas9 was extensively employed for in vivo gene editing. To ensure the efficient delivery of CRISPR/Cas9 system, small promoters such as mouse Mecp2 promoter (235 bp), miniCMV promoter (180 bp), and hybrid EF1α/HTLV (nEF) promoter (493 bp) have been used to express the widely used *Streptococcus pyogenes* Cas9 (SpCas9, ~ 4.2 kb) in one AAV vector, and the sgRNA expression cassette(s) is delivered by another AAV vector [99–101]. The discoveries of smaller Cas9 proteins from *Staphylococcus aureus* (SaCas9, ~ 3.3 kb) and *Streptococcus thermophiles* (St1Cas9, 3.5 kb) enable the packaging of sgRNA and Cas9 in a single AAV vector [102, 103]. Additionally, successful delivery of the intein-split SpCas9 was reported using a dual-AAV vector system [104]. The subsequent analyses in mouse models showed that the cleavage repaired by NHEJ at a single target site introduced indels up to 40–70% of the total alleles [100, 102], while simultaneous cleavages using two sgRNAs induced deletion of pathogenic mutations [105, 106]. Through HDR-mediated DNA replacement, a therapeutic transgene can be inserted at a pre-selected genomic site in the somatic tissues of a living organism, which can potentially be used for correcting inherited diseases [107–109].

### The expanding CRISPR toolbox prompts the development of novel strategies for gene therapy

Since the advent of engineered nucleases, researchers have been continuously developing new genome editing strategies. In 2014, Auer et al. exploited the NHEJ mechanism to capture large DNA at Cas9-induced DSB sites and established a distinct homology-independent knock-in approach [110]. In 2016, He et al. compared the NHEJ- and HDR-mediated knock-in side-by-side in various human cell types using a promoterless GFP reporter system and found that the homology-independent knock-in via NHEJ repair mechanism showed superior efficiency compared to HDR methods [111, 112]. This is consistent with the understanding that HDR is associated with DNA replication, while the NHEJ mechanism adopts a more flexible process that is largely active throughout the cell cycle [113]. In the same year, Suzuki et al. applied the homology-independent knock-in approach through AAV-mediated delivery to achieve targeted integration in mouse liver [101].

Continuous discoveries and protein engineering are rapidly expanding the CRISPR toolbox [114, 115]. Efforts in searching for new Cas9 orthologues identified a number of smaller Cas9 proteins, such as SaCas9 and St1Cas9 [102, 103], as well as Cas orthologues with higher fidelity such as Cpf1 (Cas12a) [116]. Research on rational engineering gave rise to new Cas9 variants and orthologues with greater specificity [117–119]. Investigations using protein fusion strategy developed novel gene editing tools. For instance, fusing catalytically inactivated Cas9 (dCas9) with a transcriptional activator (VP64, p65AD, SunTag, or VPR) or repressor (KRAB) generated synthetic transcription factors [120–123]. The coupling of dCas9 or mutant Cas9 (D10A) to a cytidine deaminase, such as APOBEC1 and AID, produced the base editor (BE) that can catalyze single base pair substitutions within targeted sequences [124, 125]. Collectively, the adaptation of CRISPR technology coupled with gene editing strategies unveils great potentials and prompts the development of novel gene therapy strategies [126].

## Broadening the prospects of AAV-delivered therapeutics through somatic gene editing

Undoubtedly, the advancement of in vivo gene targeting using AAV-mediated delivery of ZFN or CRISPR has propelled intensive research to develop gene editing therapies for treating deleterious inherited diseases that were previously untreatable. In the following sections, we will summarize the recent progress of AAV-based somatic gene editing used in preclinical and clinical studies (Tables 3 and 4), and discuss the inherent challenges from these in vivo studies.

**Table 3:** Targeting strategies and preclinical studies for AAV-delivered in vivo gene editing therapies

Gene editing strategy	Disease	Target tissue	Gene to be corrected	Nuclease used	AAV serotype and number of vectors used	Route of administration and age for treatment	Outcome and therapeutic effects	Ref
HDR-mediated gene correction	Hyperammonemia	Liver/hepatocyte	*Otc*	SaCas9	AAV8 2 vectors	i.v. temporal vein at P2	Correction of the mutation in 6.7–20.1% of hepatocytes; partial rescue of lethality in mice fed with high-protein diet.	[108]
	Hyperammonemia	Liver/engrafted human primary hepatocyte	*hOTC*	SaCas9	AAV (NP59) 2 vectors	i.v., tail vein in adult mice	Correction of the mutation in up to 29% of *hOTC* alleles in the engrafted primary human hepatocytes.	[127]
	Familial hypercholesterolemia	Liver/hepatocyte	*Ldlr*	SpCas9	AAV8 2 vectors	Subcutaneously injection at P1	Restored LDLR protein levels to 18.01 ± 2.82%.	[128]
	Phenylketonuria (PKU)	Liver/hepatocyte	*Pah*	SpCas9	AAV8 2 vectors	i.v., facial vein at P3	Yielded around 10% HDR-corrected reads and reduced serum phenylalanine levels	[129]
Allelic exchange through HDR	Hereditary tyrosinemia type I (HT1) and mucopolysaccharidosis type I (MPS1)	Liver/hepatocyte	*Fah* or *Idua*	SpCas9	AAV8 or AAV9 2 vectors	i.v., facial vein at P1; tail vein in adult mice	Restored FAH activity in the liver from 0.4 to 1.6% of normal. Restored IDUA activity to ∼ 0.5% of the wild-type level, and substantially reduced GAG accumulation in the heart	[130]
HDR-mediated gene knock-in	Hemophilia B	Liver/hepatocyte	*hF9* at *Rosa26*	ZFN	AAV8 2 vectors	i.p. at P2	Targeting efficiencies in the 1–3% range, and yielded 3–7% of normal circulating levels of hFIX	[96]
	Hemophilia B	Liver/hepatocyte	*hF9* at *Rosa26*	ZFN	AAV8 2 vectors	i.v., tail vein in adult mice	Treated animals exhibited long-term expression of hFIX averaging 23% of normal level (11,466,100 ng/mL) at week 60	[97]
	Hemophilia A/B	Liver/hepatocyte	*hF*8, *hF9* at *Alb* In1	ZFN	AAV8 2 vectors	i.v., tail vein in adult mice	Achieved long-term expression of hFVIII and hFIX at therapeutic levels in mouse models of hemophilia A and B, respectively	[98]
	Hemophilia B	Liver/hepatocyte	*mF9*	SaCas9	AAV8 2 vectors	i.p. at P0; i.v., tail vein in adult mice	Circulating FIX level reached to 6.013 ± 0.60%	[107]
	Hemophilia B	Liver/hepatocyte	*hF9*-Padua at *mF9*	SaCas9	AAV8 2 vectors	i.v., temporal vein at P2; tail vein in adult mice	All treated mice produced therapeutic hFIX in serum at 10.9% ± 1.6% of normal	[109]
	Crigler-Najjar syndrome type I (CN-1)	Liver/hepatocyte	*Ugt1A1* at Alb Ex14	SaCas9	AAV8 2 vectors	i.p. or i.v. retro-orbital sinus at P2–P4	Yielded 3–4% of recombinant hepatocytes	[131]
NHEJ-mediated gene knock-in	Retinitis pigmentosa	Retina/retinal pigment epithelium	*Mertk* In1	SpCas9	AAV8 2 vectors	Subretinal injection in adult rat	Yielded *Mertk* expression around 4.5% of normal	[101]
	Hemophilia A	Liver/hepatocyte	*hF8* at *Alb* In13	SaCas9	AAV8 2 vectors	i.v., tail vein in adult mice	Produced circulating FVIII at levels of 2–13% of normal and restored hemostasis.	[132]
	Hemophilia A	Liver/hepatocyte	*hF8* at *Alb* In11-13	SpCas9	AAV8 3 vectors	i.v., tail vein in adult mice	Restored hemostasis in mF8^-/-^ mice	[133]
NHEJ-based gene disruption	Cholesterol homeostasis	Liver/hepatocyte	*Pcsk9*	SaCas9	AAV8 1 vector	i.v., tail vein in adult mice	Yielded a 95% decrease of serum Pcsk9 and a 40% decrease in total cholesterol	[102]
	Hereditary tyrosinemia, type I (HTI) and type III (HT3)	Liver/hepatocyte	*Hpd*^*I335M*^	St1Cas9	AAV8 1 vector	i.v. retro-orbital sinus at P2 mice	Rescued lethality and metabolic defects in *Fah*^*-/-*^ mice	[103]
	Age-related macular degeneration (AMD)	Retina/retinal pigment epithelium cell	*Vegfa* or *Hif1a*	CjCas9	AAV9 1 vector	Intravitreal injection in 8 weeks mice	CjCas9 targeted to the Vegfa or Hif1a gene in RPE cells reduces the size of laser-induced choroidal neovascularization, suggesting that in vivo genome editing with CjCas9 is a new option for the treatment of age-related macular degeneration	[134]
	Age-related macular degeneration (AMD)	Retina/retinal pigment epithelium cell	*Vegfa* or *Hif1a*	LbCpf1	AAV9 1 vector	Intravitreal injection in adult mice	LbCpf1 targeted to Vegfa or Hif1a in RPE cells reduced the area of laser-induced choroidal neovascularization as efficiently as aflibercept, an anti-VEGF drug currently used in the clinic, without inducing cone dysfunction	[135]
	Age-related macular degeneration (AMD)	Retina/retinal pigment epithelium cell	*Vegfa*	SpCas9 and SaCas9	AAV8 2 vectors for SpCas9 1 vector for SaCas9	Subretinal injection in adult mice	Successful VEGF knockdown using AAV-mediated CRISPR systems may be a potential therapeutic strategy for CRISPR-based treatment of CNV in neovascular AMD	[136]
	Autosomal dominant cone-rod dystrophy	Retina/photoreceptor cell	*GUCY2D*	SaCas9	AAV5 1 vector	Subretinal injection in adult mice and Macaque	Reduced retGC1 expression in mouse and macaque photoreceptors in vivo, and demonstrably improved retinal function and structure	[137]
	Wolff-Parkinson-White syndrome	Heart/cardiomyocytes	*Prkag2*^*H530R*^	SpCas9	AAV9 2 vectors	i.c.v. injection at P4; i.v. in adult mice	About 20% reduction in the *Prkag2* mutant mRNA level in mice receiving AAV injection on P4 or P42	[138]
	Retinitis pigmentosa	Retina/retinal pigment epithelium	*Rho*^+/P^23^H^	SpCas9-VQR	AAV9-PHP.B 2 vectors	Intravitreal injection in adult mice	Effective disruption of the P23H *Rho* mutant	[139]
	Hearing loss	Ear/sensory hair cell	*Tmc1*	SaCas9-KKH	AAV2/Anc80 1 vector	Inner-ear injection at P1	Detection of 0.2%, 1.8%, 1.6%, and 2.2% indel frequencies at 7, 14, 42, and 55 days after injection, respectively	[140]
	HIV-1	T cell	HIV-1 proviral DNA	SaCas9	AAV-DJ/8 1 vector	i.v., tail vein in adult mice	In vivo excision of HIV-1 proviral DNA by sgRNAs/saCas9 in solid tissues/organs can be achieved via AAV delivery	[141]
	HIV-1	T cell	HIV-1 proviral DNA	SaCas9	AAV 9 1 vector	i.v., tail vein in adult mice	Eradication of HIV-1 in infectious cell and tissue sites of infected animals was achieved	[142]
NHEJ-based deletion of mutations (or exon skipping)	Leber congenital amaurosis (LCA)	Retina	*CEP290* (IVS26)	SpCas9	AAV 5 2 vectors	subretinal injection in adult mice	NGS analysis of four treated retinas revealed that 7.5%, 21.1%, 26.4%, and 25.2% of sequences comprised truncated DNA following targeted genomic deletion	[143]
	Duchenne muscular dystrophy (DMD)	Muscle	*Dmd* Ex23	SpCas9	AAV9 2 vectors	i.p.at P1; i.m. at P12; i.v. retro-orbital sinus at P18	Partially restored dystrophin protein expression in cardiac and skeletal muscles	[99]
	DMD	Skeletal and cardiac muscle	*Dmd* Ex23	SaCas9	AAV9 2 vectors	i.m. in adult mice; i.p.at P3; i.v., tail vein in adult mice	Restored dystrophin expression in 3–18% myofibers, cardiomyocytes, and muscle stem cells after local or systemic delivery	[105]
	DMD	Skeletal and cardiac muscle	*Dmd* Ex23	SaCas9	AAV8 2 vectors	i.p. at P2 i.v., tail vein in adult mice	Partial recovery of functional dystrophin in skeletal myofibers and cardiac muscle; improvement of muscle biochemistry and muscle force	[106]
	DMD	Muscle	*Dmd* Ex23	SaCas9	AAV8,AAV9 2 vectors	i.m. and i.v. facial-vein at P2 i.m. in adult mice	Sustained restoration of dystrophin expression in *Mdx* mouse model for 1 year	[144]
	DMD	Cardiac muscle	*Dmd* Ex23 (excision Ex21-23)	SaCas9	AAVrh.74 2 vectors	i.p. at P3 mdx mice	Restored dystrophin expression and improved cardiac function	[145]
	DMD	Skeletal and cardiac muscle	*Dmd* Ex44 (excision Ex44-45)	SpCas9	AAV9 2 vectors	i.p. at P4	Partial restoration of dystrophin expression and improved muscle contractility	[146]
	DMD	Skeletal and cardiac muscle	*Dmd* Ex51 (excision Ex51-52)	SpCas9 intein-split	AAV9 2 vectors	i.m at P10–14 in pigs	Expression of a shortened dystrophin (DMDΔ51–52) and improved skeletal muscle function	[147]
	Muscular dystrophy type 1A (MDC1A)	Skeletal muscle	*Lama2* Ex2	SaCas9	AAV9 2 vectors	i.p. at P2 i.m. in adult mice	Inclusion of exon 2 in ~ 20% of Lama2 transcripts	[148]
Base editing	DMD	Muscle	*Dmd* Ex20	ABE	AAV9 2 vectors	i.m. in adult mice	Restored dystrophin expression in 17 ± 1% of myofibers	[149]
	Hereditary tyrosinemia type 1 (HT1)	Liver/hepatocyte	*Fah*	ABE7.10	AAV8 2 vectors	i.v., tail vein in adult mice	Desired conversion was achieved in 29.1–33.4% of total sequencing reads in the high-dose group 2 months after AAV injection	[150]
	Phenylketonuria (PKU)	Liver/hepatocyte	*Pah*	nSaKKH-BE3	AAV8 2 vectors	i.v., tail vein in adult mice	*Pah* mRNA correction rates reached 39.1–47.1% at 26 weeks post injection	[151]
	Amyotrophic lateral sclerosis (ALS)	Spinal cord/astrocytes	*Sod1*	CBE	AAV9 2 vectors	Spinal injection in adult mice	Average 1.2% of the analyzed reads from mice had the CBE-introduced mutations	[152]
Guided gene silencing or activation	Cholesterol homeostasis	Liver/hepatocyte	*Pcsk9*	dSaCas9KRAB	AAV8 2 vectors	i.v., tail vein in adult mice	Yielded up to 90% repression of Pcsk9 levels	[153]
	Dravet syndrome	Parvalbumin interneuron	*Scn1a*	dCas9-VP64	AAV9 2 vectors	Intracerebroventricular injection at P0	Recovered the firing ability of parvalbumin interneurons, and attenuated febrile seizures	[154]

**Table 4:** Clinical studies for AAV-delivered gene editing therapies

Product name	Date of IND approval, agency	Manufacturer	Disorder/condition	Target tissue	AAV vector	Gene editing tool	Gene to be delivered	Targeting strategy and locus	Clinical trials	Status/clinical outcome	Ref
SB-FIX	Dec 2015, FDA	Sangamo Therapeutics	Hemophilia B	Liver, in vivo	ssAAV6	ZFN	*hF9*	Targeted insertion at *ALB* locus	NCT02695160	Entered phase 1 clinical trial, with the first patient dosed in Dec 2018	-
SB-318	Feb 2016, FDA	Sangamo Therapeutics	Mucopolysaccharidosis type I (MPS I)	Liver, in vivo	ssAAV6	ZFN	*IDUA*	Targeted insertion at *ALB* locus	NCT02702115	Entered phase 1/2 clinical trial, with the first patient dosed in Dec 2018	[155]
SB-913	June 2016, FDA	Sangamo Therapeutics	Mucopolysaccharidosis type II (MPS II)	Liver, in vivo	ssAAV6	ZFN	*IDS*	Targeted insertion at *ALB* locus	NCT03041324	Entered phase 1/2 clinical trial, with the first patient dosed in Nov 2017	[156]
EDIT-101 (AGN-151587)	Nov 2018, FDA	Editas Medicine	Leber Congenital Amaurosis type 10 (LCA10)	Retina, in vivo	ssAAV5 1 vector	SaCas9 sgRNAs	-	Deleting IVS26 mutation in CEP290 In26 via dual-cut targeting	NCT03872479	Entered phase 1/2 clinical trial, with the first patient dosed on March 2020	[157]

### Correcting genetic mutations by HDR-mediated sequence replacement

AAV-CRISPR-mediated DNA replacement via the HDR mechanism has the potential to correct pathogenic mutations in somatic genome. The correction will then remain stable in the genome even in dividing cells, making the genetic correction strategy a suitable treatment option for neonates. Yang et al. carried out a proof-of-concept study by infusing AAV vectors carrying SaCas9, sgRNA, and HDR donor template into neonatal spf-ash mice, which carries the R129H mutation in *Otc* gene and exhibited a partial deficiency in the urea cycle enzyme ornithine transcarbamylase (OTC) [108]. The study demonstrated the correction of the mutant *Otc* gene in 10% of the total hepatocytes, as well as increased survival of mice challenged with a high-protein diet [108]. Using a similar approach but targeting human hepatocytes in mouse models with human hepatocyte engraftment, Ginn et al. achieved targeted correction among 29% of human *OTC* alleles in the patient-derived primary hepatocytes [127]. Similarly, Zhou et al. performed subcutaneous injection to deliver AAV vectors carrying SpCas9, sgRNA, and donor into neonatal *Ldlr*^*E208X*^ mice that harbors a nonsense mutation E208X in the *Ldlr* gene to mimic familial hypercholesterolemia (FH). The treatment significantly ameliorated atherosclerosis in mice fed with a high-fat diet [128]. In another study, newborn *Pah*^*enu2*^ mice, a disease model for phenylketonuria (PKU), intravenous delivery of AAV vectors carrying SpCas9, sgRNA, and donor DNA yielded significant correction of the mutations as well as reduction of serum phenylalanine levels [129].

Other than providing a donor template for HDR-based mutation correction, Wang et al. reported that inducing cuts simultaneously at two alleles of a gene could also trigger inter-homolog translocation and allelic exchange, thereby correcting recessive compound heterozygous mutations through the HDR mechanism. By introducing genome cuts into newborn mice, their study showed allelic exchange and disease rescue in *Fah*^*neo/PM*^ and *Idua*^*neo/W392X*^ mice to mimic the disease conditions for hereditary tyrosinemia type I (HT1) and mucopolysaccharidosis type I (MPS I), respectively [130].

### Targeted insertion of therapeutic sequences in somatic genome

Given the huge diversity of loss-of-function mutations that may occur within a single disease-related gene, targeted DNA insertion at a defined locus is a more direct strategy to restore gene function. Inherited hemophilia B has been used as a study model for testing various targeted integration strategies for gene therapy. Li et al. and Anguela et al. used HDR-based replacement strategy to introduce targeted insertion of *hF9* gene exon (*hF9* Ex2-8) at a mutant *hF9* transgene locus in mouse liver using AAV-delivered ZFN and reported successful production of functional hFIX as well as the reversal of blood coagulation defect in hemophilia B mice [96, 97]. By using the AAV-delivered SaCas9, Ohmori et al. inserted the normal *mF9* gene Ex2-8 [107]*,* and Wang et al. knocked-in the Ex2-8 of the hyperactive *hF9* variant (hFIXco-Padua), at the mutant *mF9* locus [109]. Both studies achieved successful gene targeting, stable production of circulating FIX, and significant improvement of hemophilia B-related symptoms [107, 109]*.*

Much effort was also focused on surveying general target sites that can potentially be used for different therapeutic targeting. Through AAV-ZFN-mediated HDR-replacement, Sharma et al. showed that targeted insertion at intron 1 of Albumin locus (*mAlb* In1) could achieve long-term expression of human FVIII and FIX at therapeutic levels [98]. By targeting the same site, the group also successfully produced lysosomal enzymes encoded by GAL, GBA, IDUA, and IDS, which are responsible for Fabry and Gaucher diseases as well as Hurler and Hunter syndromes [98]. Likewise, De Caneva et al. successfully rescued neonatal lethality in mice with Crigler-Najjar syndrome by inserting *hUGT1A1* at *mAlb* Ex14 through AAV- and SaCas9-mediated HDR [131]. Together, these studies suggest that the *ALB* locus could be a potential universal target locus for targeted insertion to express liver secretory proteins.

Homology-independent knock-in mediated by the NHEJ mechanism does not require homology sequences, which makes AAV delivery much easier. By using SpCas9, Suzuki et al. demonstrated the potential of AAV-delivered homology-independent targeted insertion (also named HITI) to restore *Merk* gene expression and function in the rat retina, which successfully ameliorated visual impairment associated with retinitis pigmentosa [101]. Using AAV-delivered SaCas9, Chen et al. and Zhang et al. reported NHEJ-mediated insertion of BDD-FVIII at *mAlb* In13 [132] and multiple sites within *mAlb* In11-13, respectively, which restored FVIII production and hemostasis in mice with hemophilia A [133].

In contrast to AAV-based gene augmentation therapy, gene editing therapy via targeted integration could utilize promoterless donors. Thus, the therapeutic sequences will only be expressed upon correct insertion. Moreover, a well-characterized targeting site can be established to support the insertion and expression of various therapeutic genes in a tissue-specific manner. These new features could further reduce the risks of unwanted gene activation or uncontrolled expression.

### Gene (allele) disruption via site-specific targeting followed by NHEJ repair

The versatile AAV-delivered nucleases can also generate frameshift mutations via NHEJ repair in a target gene in the somatic genome to disable the translation of defective proteins. By targeting a cholesterol regulatory gene *Pcsk9* in mouse liver using AAV8 encoding SaCas9, Ran et al. introduced indels at around 40% of the target alleles, which significantly reduced serum PCSK9 and total cholesterol levels [102]. Likewise, intravitreal delivery of AAVs carrying CjCas9 [134] and LbCpf1 [135] successfully disrupted angiogenesis-associated genes *Vegfa* and *Hif1a* in the mouse retina, which substantially decreased the excessive choroidal neovascularization in a transgenic model for age-related macular degeneration (AMD) [134, 135]. More recently, Chung et al. also demonstrated the therapeutic potentials of AAV-delivered SaCas9 and SpCas9 to treat AMD [136]. Markedly, the AAV-CRISPR-mediated in vivo gene disruption also resolved the gain-of-function mutations in the photoreceptor guanylate cyclase (*GUCY2D)* gene that causes dominant cone-rod dystrophy (CORD6) in nonhuman primate (NHP) [137].

A sgRNA can also be programmed for allele-specific disruption. In an effort to correct autosomal dominant inherited diseases, Xie et al. disrupted the mutant allele encoding PRKAG2^H530R^ using AAV-delivered SpCas9 while sparing the wild-type allele intact, resulting in successful correction of PRKAG2^H530R^-induced cardiac syndrome in the transgenic mouse model [138]. Similarly, Giannelli et al. corrected Retinitis Pigmentosa caused by the dominant Rho^P23H^ mutation in mice [139]; and Gyorgy et al. prevented deafness in mice carrying the *Beethoven* mutation (TMC1^T1253A^) that causes degeneration of cochlear hair cells and progressive hearing loss [140].

Gene editing using AAV-delivered CRISPR/Cas9 has also been explored as antiviral therapeutics and tested for treating chronic viral infections, such as human immunodeficiency virus (HIV). Intravenous injection of AAV vectors carrying multiplexed sgRNAs and SaCas9 was shown to induce proviral excision, and subsequently decrease viral gene expression in the HIV-1 Tg26 transgenic mice and humanized mice with chronic HIV-1 infection [141]. More recently, the combined application of sequential long-acting slow-effective release antiviral therapy (LASER ART) and CRISPR/Cas9 showed great potential in complete eradication of HIV in HIV-1-infected humanized mice [142].

### Restoring gene function through dual-sgRNA-directed deletion

AAV-mediated delivery of Cas9 with dual sgRNAs targeting both sides of a sequence could induce targeted deletion via NHEJ repair and has been exploited to eliminate deleterious mutations, such as cryptic splice sites that cause severe splicing errors. Leber congenital amaurosis-10 (LCA10) is an inherited retinal dystrophy that is often attributed to IVS26 mutation in the CEP290 gene which creates a de novo splice donor site and produces transcripts with a premature stop codon [158]. Using AAV5-delivered SpCas9 and carefully selected dual sgRNAs, Ruan et al. excised the IVS26 mutation in mouse photoreceptor cells, which restored the splicing and function of CEP290 and rescued vision loss [143]. Later on, Maeder et al. developed a gene editing therapy based on subretinal delivery of SaCas9 and two sgRNAs in a single AAV5 vector, named EDIT-101, and demonstrated successful excision of the IVS26 containing region and substantial restoration of the CEP290 function in humanized CEP290 mice [157]. Subsequently, a surrogate vector also achieved successful editing of the CEP290 gene and demonstrated therapeutic benefits in NHP [157]. With these efforts, Allergan and Editas Medicine launched their landmark phase 1/2 clinical trial for EDIT-101 and commenced dosing in the first LCA10 patient in March 2020, which sets the record to be the first in vivo CRISPR medicine administered to patients with FDA approval [159].

A similar therapeutic strategy is also intensively tested for treating Duchenne muscular dystrophy (DMD), the most common form of muscular dystrophy caused by mutations in the *DMD* gene. Based on the clinical evidence that exon skipping in DMD patients is associated with milder symptoms, research groups first applied the dual-sgRNA strategy to excise exon 23 carrying a nonsense mutation. Studies using *mdx* mice achieved successful deletion of the mutant Ex23 in both neonatal and adult mice, which yielded shorter yet partially functional dystrophins and significantly improved muscular organizations and functions [99, 105, 106, 144]. Other groups have also used the dual-sgRNA strategy to excise longer genomic regions, such as *Dmd* Ex21-23 or Ex44-45, to restore muscle function in the *mdx* mice [145, 146]. Moreover, intramuscular injection of AAV9 carrying intein-split SpCas9 to excise *DMD* Ex51-52 in DMD pigs also improved their skeletal muscle function [147]. Interestingly, Dwi et al. excised *Lama2* Ex2 to eliminate an aberrant splicing donor site causing congenital muscular dystrophy type 1A (MDC1A), which partially restored muscle function in the *dy2J/dy2J* mouse model [148].

### Base editing approach for gene correction and knockout

Base editing is a novel DNA-engineering approach which enables programmable base-substitutions in the genome for correcting pathogenic point mutations. Base editors (BEs) are generated by fusing mutant Cas9 with a cytidine or adenine deaminase [124, 125]. Despite the large sizes of the BEs, in vivo delivery was achieved using dual AAV vector systems [104]. By using the intein-split approach, Ryu et al. demonstrated the therapeutic potential of adenine base editors (ABEs) in correcting a nonsense mutation in the *Dmd* gene [149]. Yang et al. applied AAV-delivered cytidine base editor (CBE) to restore the start codon of the mutated *Fah* gene, which yielded functional expression in mouse liver and ameliorated the HT1 symptoms [150]. More recently, Levy et al. reported in vivo base editing at therapeutically relevant efficiencies in a broad range of mouse tissues, including brain (up to 59%), liver (38%), retina (38%), heart (20%), and skeletal muscle (9%) [160]. Through trans-splice strategy, Villiger et al. corrected the *Pah*^*enu2*^ c.835 T > C mutation through intravenous injection of AAVs carrying split CBE, which subsequently restored PAH enzyme activity and serum phenylalanine levels in *Pah*^*enu2*^ mice [151].

BEs are also used to introduce nonsense mutations to inroduce a loss-of-function effect [161]. CBE delivered via adenovirus vector has been tested to reduce the *Pcsk9* and *Hpd* expressions in the mouse model with inherited hypercholesterolemia [162]. In another study, AAV-mediated intein-split delivery of CBE was implemented to disable the mutant *SOD1* allele in SOD1^G93A^ mice, which markedly slowed down the progression of amyotrophic lateral sclerosis (ALS) disease and prolonged survival [152].

### Feasibility of gene editing therapy in neonate and fetus

In vivo genome editing has presented a promising potential for early gene intervention in neonates or fetuses to treat previously untreatable diseases. Intraperitoneal (i.p.) injection is the most frequently used route of administration and supports the delivery of CRISPR/Cas9 reagents into murine pups at postnatal day 1–4 to correct the congenital or Duchene muscular dystrophy [99, 106, 148]. Intravenous injection (i.v.) via facial or temporal veins is also widely used in neonatal pups and achieved gene editing in the liver to correct metabolic disorders, such as hyperammonemia [108], PKU [129], and hypercholesterolemia [128] which are caused by genetic defects in the *Otc*, *Pah*, and *Ldrl* genes, respectively. Local injections into the muscles or in the cochlear of neonatal pups have also tested and achieved successful somatic gene editing to correct DMD and hearing loss, respectively [99, 140]. The possibility to perform therapeutic gene editing before birth was also examined, wherein in utero editing of *Pcsk9* and *Hpd* genes confirmed the long-term persistence of edited cells in postnatal mice [162]. Collectively, these studies support the feasibility of using gene editing intervention in fetuses and neonates to correct defective genes before disease onset, which is critical for treating diseases with high morbidity and mortality.

## Combined delivery of AAV-donor and non-viral CRISPR/Cas9 for ex vivo gene editing therapy

In earlier studies, the AAV vector was rarely used for transducing hematopoietic stem and progenitor cells (HSPC) because the rapid cell proliferation in the subsequent differentiation processes will quickly dilute the vectors and abolish transgene expression. Soon after the advent of engineered nucleases, studies found that AAV6 transduces HSPC with high efficiency and provides single-stranded DNA as donor template, which enables superior HDR-based gene editing when co-delivered with ZFN mRNA or Cas9/sgRNA ribonucleoprotein (RNP) [163–165]. As a result, there has been rapid development of ex vivo gene editing therapeutics for treating HSPC-based inherited diseases (Table 5).

**Table 5:** Targeting strategies for ex vivo gene editing

Gene editing strategy	Disease	Target cells	Gene to be corrected	Nuclease used	HDR donor	Purposes and results	Ref
HDR-mediated gene knock-in	-	HSPC	CCR5 and AAVS1	ZFN (mRNA)	AAV6 1 vector	Achieved site-specific insertion of GFP cassette at the CCR5 and AAVS1 loci, in up to 26% of adult HSPCs and up to 43% of fetal liver HSPCs	[164]
	-	HSPC and T cell	CCR5	SpCas9 (RNP)	AAV6 2 vectors	Achieved targeted insertion of large transgene cassettes in primary human cells using HR-mediated genome editing with AAV vectors	[165]
HDR-mediated gene correction	X-linked severe combined immunodeficiency (SCID-X1)	HSPC	IL2RG	ZFN (mRNA)	Integrase-defective lentiviral vector (IDLV)	Achieved targeted gene correction in ~ 6% of HSPCs, for treating SCID-X1	[166]
	Sickle cell disease (SCD)	HSPC	HBB^E6V^	SpCas9 (RNP)	AAV6 1 vector	Achieved targeted correction of ~ 50% of the HBB^E6V^ alleles in HSPCs derived from SCD patients	[163]
	SCD	HSPC	HBB^E6V^	SpCas9 (RNP)	ssODN	Achieved gene correction for up to 25% of the HBB^E6V^ alleles in HSPCs derived from SCD patients	[167]
	SCID-X1	HSPC	IL2RG	SpCas9 (RNP)	AAV6 1 vector	Achieved targeted gene correction in ~ 45% of HSPCs from six SCID-X1 patients, and rescued lymphopoietic defect in vitro and in vivo	[168]
HDR-based gene disruption	Cancer	T cell	TRAC	SpCas9 (RNP)	AAV6 1 vector	HDR knock-in of the CAR into the TRAC locus to generate TCR-negative CAR-T cells	[169]
	Cancer	T cell	TRAC and PD-1	LbCpf1 (mRNA)	AAV6, AAV9 1 vector	Produced CAR-T cell with HDR knock-in and immune-checkpoint knockout (KIKO CAR-T cell) in one step	[170]
NHEJ-based gene disruption	β-Thalassemia	HSPC	Erythroid enhancer in HBG1/2 locus, at − 102 to − 115 bp upstream of the TSS	SpCas9 (lentivirus)	-	Edited HSPC-produced RBCs with increased HbF levels that inhibited the pathological hypoxia-induced RBC morphology found in SCD	[171]
	β-Thalassemia	HSPC	13 kb of the β-globin locus	SaCas9 (nucleofection)	-	Achieved targeted deletion in 31% of HSPCs. The erythroid colonies with the targeted deletion showed significantly higher γ-globin expression	[172]
	β-Thalassemia	HSPC	HBG1/2 gene promoter, at − 115 and − 200 bp upstream of the TSS	SpCas9 (nucleofection)	ssODNs or plasmids	Introduced deletions, disrupted repressor binding and raised γ-globin gene expression	[173]
	β-Thalassemia	HSPC	13 kb of the β-globin locus	SpCas9 (nucleofection)	-	Resulted in a high γ-globin expression in erythroblasts, increased HbF synthesis, and amelioration of the sickling cell phenotype	[174]
	HIV	HSPC	CCR5	ZFN (mRNA)	-	Disrupted *CCR5* gene in around 17% of the total alleles in human HSPC, significantly lowered HIV-1 levels	[175]
	HIV	HSPC	CCR5	ZFN (mRNA)	-	Achieved biallelic *CCR5* disruption in up to 72.9% of modified colony-forming units derived from adult HSPC	[176]
	HIV	HSPC	CCR5	SpCas9 (RNP)	-	Achieved efficient *CCR5* ablation in long-term HSPCs, which confers HIV-1 resistance in vivo in mouse model	[177]
	HIV	HSPC	CCR5	SpCas9 (RNP)	-	Achieved disruption of *CCR5* gene in 5% of lymphocytes; has entered clinical trial (NCT03164135)	[178]

Ex vivo gene editing in HSPCs provides an ideal strategy for treating inherited hemoglobinopathies and immunodeficiencies, such as sickle cell disease (SCD), β-thalassemia, and X-linked severe combined immunodeficiency (SCID-X1). Earlier studies used ZFN mRNA and integrative deficient lentivirus vector (IDLV) to demonstrate the HDR-based targeted insertion of a corrective cDNA into the *IL2RG* locus of the HSPCs isolated from SCID-X1 patient [166]. Using Cas9/sgRNA RNP and oligonucleotide donors, Dewitt et al. corrected the sickle cell anemia mutation HBB^E6V^ in the HSPCs isolated from SCD patients [167]. More strikingly, the co-administration of AAV6 donor and Cas9/sgRNA RNP for HDR-based gene editing in HSPCs derived from SCD patients achieved significantly higher rates for the correction of sickle mutation E6V and targeted insertion. The edited HSPCs were then transplanted into immunodeficient mice and restored normal β-globin expression in vivo [163]. Additionally, Pavel-Dinu et al. reported the correction of X-SCID and long-term engraftment of the edited HSPCs, which provided substantial preclinical evidence supporting the therapeutic potential of using ex vivo editing for X-SCID [168].

Studies also transduced HSPC with Cas9/sgRNA RNP alone to introduce NHEJ-based gene disruption, and applied this strategy to abolish the repression of γ-globin genes *HBG1/2* in HSPCs ex vivo for the treatment of SCD and β-thalassemia. The results showed that disruption of the repressor binding motif in the *HBG1/2* loci or the β-globin gene *HBB* locus could re-activate γ-globin expression to ameliorate disease symptoms [171–174]. Markedly, the CRISPR-disruption of *BCL11A* enhancer developed by CRISPR Therapeutics and sponsored by Vertex Pharmaceuticals, named CTX001, has been approved as the first human CRISPR trial for SCD (NCT03745287) and transfusion-dependent β-thalassemia (NCT03655678).

The HSPC-based ex vivo gene editing has also provided an appealing treatment strategy for HIV infection. HIV attacks human T cells through binding to the CCR5 receptor [179]. Hence, ex vivo targeting of the CCR5 gene in HSPCs followed by autologous transplantation could potentially provide treatment by preventing HIV entry into the edited HSPCs [164, 175]. Studies using various gene delivery methods have yielded consistent results, showing that the CCR5-ablated human HSPCs indeed conferred HIV-1 resistance in mouse models [175–177]. These promising preclinical outcomes are currently being tested in several clinical trials [178].

Besides HSPCs, ex vivo gene editing also presents enormous potentials to engineer immune cells, especially the revolutionary immunotherapy using chimeric antigen receptor (CAR) T cells (CAR-T). Targeted insertion of anti-CD19 CAR at *TRAC* locus through co-administration of AAV6 donor and Cas9/sgRNA RNP demonstrated a stable CAR expression and improved effectiveness in the CAR-modified T cells [169]. Moreover, the *TRAC* gene disruption enables allogeneic transplantation, which permits the generation of “universal” CAR-T cells from healthy donors and supports the manufacturing of off-the-shelf CAR-T products [169]. Dai et al. used a similar strategy to insert the CAR at different immune-modulating gene loci, such as *B2M*, *CD52*, *HLA-1*, and generated CAR-T cells with immune-checkpoint knockout (KIKO CAR-T cell) [170]. Compared to lentivirus transduction, the targeted CAR insertion via AAV6-Cas9 RNP co-delivery significantly reduced random integrations as well as chances of undesired side effects in the CAR-T cells. As a result, the new CAR-T cells generated through AAV6-Cas9 RNP using multiplex gene editing by CAR insertion and disruption of *TRAC* or other immune-modulating genes are widely explored for treating various cancers [180].

## Challenges for developing AAV-CRISPR therapy for clinical application

Accumulating preclinical studies and ongoing clinical trials using the AAV delivery system have unveiled new challenges for the development of AAV-CRISPR-based gene editing for clinical application.

### Integration of AAV vectors at DSB sites of the genome

The AAV vector genomes mainly persist in transduced cells as episomes, while sporadic integrations through DNA DSB capture have been observed [181]. The likelihood for AAV integration to occur is around 0.05% in neonatal mice and between 10E−4 and 10E−5 in the liver and muscle of nonhuman primates and humans [182, 183]. The integration of AAV vector sequences poses risks of insertional mutagenesis, but the tumorigenic potentials in clinical applications remain controversial. Studies in mice have reported an increased incidence of hepatocellular carcinoma (HCC) that was attributed to random AAV integration that inadvertently activated the transcription of oncogenes [184, 185]. On the contrary, other studies provided evidence supporting that the non-integrative nature of AAV does not pose an increased risk for cancer development. Bell et al. conducted a thorough histology analysis of 695 mouse subjects [186] and Li et al. reported an 18-month follow-up of the mouse subjects [187], after AAV-based gene treatment. Both studies reported no correlation between tumorigenesis and AAV-based gene delivery. Similar investigations were conducted in dogs, NHPs, and human patients, which also showed no associated risk for AAV vector-induced malignancy [188, 189]. Furthermore, several studies also examined the correlation between wtAAV integrations and the occurrence of human hepatocellular carcinoma using clinical samples, which reported contradictory findings and remained inconclusive [190, 191].

Another potential issue related to gene editing therapy using AAV vectors is whether CRISPR and other nucleases will increase the incidence of integration events. A recent study by Nelson et al. showed that, while AAV-CRISPR genome editing in *mdx* mice exhibited sustained restoration of dystrophin function, unintended genome alterations such as the AAV integrations at sgRNA target sites were detected without apparent consequences on the mice [144]. Similarly, through genome-wide mapping of the mouse brain DNA after stereotactic injection of AAV vectors, a high level of AAV integration with strong preference at the target sites was observed [192]. In both studies, the low levels of random AAV integrations were detected throughout the genome, which was not associated with the CRISPR editing and did not pose a risk higher than that introduced by wtAAVs in humans [144, 192]. Collectively, further investigation should be directed to improve targeting strategies to minimize integration-induced mutagenesis, while the consequences of the high-level AAV integration at specific target sites may need to be evaluated on a case-by-case basis.

### Off-target effects in genome editing with CRISPR systems

The sgRNA in CRISPR system can tolerate minor mismatches to guide DNA cleavage at an off-target site [193]. Clinically, the CRISPR/Cas mismatch tolerance raises safety concerns and prompts research groups to explore ways to improve targeting specificity [194]. New algorithms are continuously generated to facilitate the selection of sgRNA with high gene editing activity and fidelity [195, 196]. Additionally, new Cas9 orthologues and engineered Cas9 variants, such as the enhanced specificity SpCas9 (eSpCas9), high fidelity SpCas9 (SpCas9-HF1) [117, 118], and the high fidelity SaCas9 (SaCas9-HF) [119], have achieved greater targeting specificity without sacrificing gene editing activity [114, 115]. Moreover, the shortened expression of Cas9 through lipid nanoparticle delivery of mRNA has greatly reduced off-target editing [197].

Extensive efforts have been made to map out off-target events at the genome level, which were later found to be challenging with the technologies that are currently available. Performing whole-genome sequencing (WGS) for edited cells is prohibitively expensive for identifying rare but potentially deleterious off-target events [198]. Gene analysis by targeted deep sequencing is limited by the sequence homology assumptions inherent in the computational prediction of potential off-target sites. To address these challenges, multiple new analysis platforms have been developed to detect Cas9 off-target events throughout the genome [194]. These include DISCOVER-Seq [199], GUIDE-seq [200], BLESS [201], CIRCLE-seq [202], and SITE-seq [203]. However, each of these platforms can only identify a portion of off-target events, and none of which can deliver a comprehensive characterization of off-target modifications to evaluate the overall functional impact. In future, multiple analysis technologies may be used together for identifying off-target events and safety assessment of a newly developed gene editing therapy.

### Risk of horizontal and vertical transmission

In clinical studies, human subjects who received systemic administration of AAV vectors were found to carry AAV particles in bodily fluids, such as serum and urine, for several weeks [8, 204]. Since AAV vectors are replication-defective, the risk for horizontal transmission is low and mainly restricted during vector transfer. However, the persistence of AAV particles with broad tissue tropisms in bodily fluids [205] makes it difficult to target specific tissues or organs without diffusing into other tissues in the human body. Consequently, AAV treatments could result in gene expression or genome editing in non-targeted tissues and potentially give rise to pathological features, which should be evaluated thoroughly.

In AAV-based gene editing therapy, vertical transmission of AAV vectors and germline contamination poses a much more serious concern [206]. Earlier studies detected AAV sequences in human semen samples [207], and a similar observation was reported in the murine testis as confirmed by the presence of AAV-delivered reporter expression [208]. Interestingly, although AAV vector sequences were detected in seminal fluids and epithelial cells from genitourinary tracts, they were not found in germ cells and had not passed through germline transmission [209, 210]. Furthermore, after a close investigation, Rajasekaran et al. reported that AAV2 and AAV9 vectors primarily targeted Leydig cells while a modified-AAV2 targeted Sertoli cells of the testis [211]. Notably, none of these vectors transduced sperm progenitor cells [211]. Consistently, in vivo gene targeting studies found no evidence of genome editing in the sperms or offspring derived from the edited mice [144]. Together, these results eliminate the concern regarding the vertical transmission of AAV and whereby induced germline modifications through systemic administration.

### Host immune responses to CRISPR and Cas proteins

Host immune responses to CRISPR/Cas9 and its orthologues may present another challenge to the development of gene editing therapy. In a recent analysis of human serums, a large proportion of the human populations was found to carry naturally occurring antibodies to Cas9. Manno et al. reported that 79% of the examined individuals exhibited anti-SaCas9, and 65% had anti-SpCas9 [212]. Wagner et al. reported 96% of the donors in their study showed pre-existing T cell memory responses against SpCas9 [213]. Consequently, the pre-existing antibody could neutralize Cas9 and impair the gene editing efficiency, while pre-existing anti-Cas9 lymphocytes can trigger immune destruction of edited cells due to Cas9 expression [214].

In order to circumvent the host immune responses to CRISPR/Cas system, it is critical to adjust the delivery parameters and strategies, such as lowering the dosage of vectors, optimizing the vector administration route, and shortening the expression of the Cas9 gene. Previously, transient immune suppression made AAV-based gene therapy possible by protecting AAV-transduced cells from the immune responses [10, 21, 215]. Therefore, it is of interest to investigate the potential of using transient immunosuppression to protect Cas9-expressing cells from immune destruction and to sustain the efficacy of gene editing. Additionally, prescreening for the presence and levels of neutralizing antibodies in patients could be implemented to determine the suitability of a treatment regimen as well as to guide the use of AAV vector capsids and Cas9 variants for each individual patient prior to treatment. Furthermore, developing a novel Cas9 variant via protein engineering to overcome the immune barriers is also worth investigating in future.

## Expanding horizons for gene therapy

### Non-viral delivery of CRISPR

Recent advancements in drug delivery using synthetic nanoparticles (NPs) have made significant progress. In 2018, a siRNA drug packaged in nanoparticles, marketed under the brand name Onpattro (patisiran), was approved by the FDA for the treatment of hereditary amyloidogenic transthyretin (hATTR, also named ATTRv) amyloidosis [216]. Extensive studies have investigated the potentials of utilizing NPs for CRISPR/Cas9 delivery, among which lipids and lipid-like nanomaterials are potentially suitable for intracellular delivery of genome editing cargos under in vivo conditions [217]. Yin et al. packaged Cas9 mRNA in lipid NPs and used in combination with AAV vectors encoding sgRNA and HDR template to induce gene correction in the *Fah*^*-/-*^ mice [197]. Lee et al. applied gold nanoparticles conjugated to DNA and complexed with cationic endosomal disruptive polymers (named CRISPR-Gold) to deliver CRISPR RNPs and donor template to correct DMD mutation in Dmx mice [218]. To maximize the delivery efficiency, Jiang et al. further developed lipid-like nanoparticles to carry Cas9 mRNA and sgRNA to the liver and demonstrated effective disruption of endogenous *Pcsk9* gene, as well as pre-delivered HBV DNA in mice [219]. In the November 2020, Intellia Therapeutics dosed the first patient with a lipid nanoparticles (LNPs)-carried CRISPR/Cas9-gene editing therapy to treat hATTR, which becomes the first systemically delivered CRISPR-based drug candidate tested in clinical trials (NCT04601051)[220]. Currently, there are still several obstacles associated with the therapeutic application of non-viral delivery for gene editing therapy; these include short half-life in the systemic circulation, non-specific delivery, and low accumulation in target tissues. Despite these limitations, the non-viral delivery methods provide invaluable additions to the gene delivery arsenal.

### Recent advances in CRISPR technologies

The research on CRISPR technology and its applications is on the rise, with new tools and strategies being developed and tested continuously. Primer editors (PEs) are a newly developed gene editing tool, which consists of a chimera PE protein generated through fusing mutant Cas9 (H840A) with a reverse transcriptase and a primer editing guide RNA (pegRNA) that carries desired genetic information and recognizes a target sequence via base-pairing. Together, the PE/pegRNA complex can directly write new genetic information into a specified DNA site [221]. Using the prime editing technology, Anzalone et al. achieved targeted insertions, deletions, and all 12 types of point mutations in human cells, including the correction of mutations that cause sickle cell disease and Tay-Sachs disease [221].

Distinctly, the Cas orthologues in the Cas13 family possess unique properties for RNA editing, which enables a novel RNA editing approach for gene therapy [222]. Compared to RNA interference (RNAi) for RNA editing, the RNA targeting by CRISPR/Cas13 triggers targeted degradation of a selected RNA with high specificity [223, 224]. Lipid-based delivery of Cas13a protein and guide RNA has been used for in vivo experiments to target a mutant KRAS transcript and was found to effectively impair tumor proliferation of pancreatic cancer in a xenograft mouse model [225]. In early 2020, CRISPR/Cas13-based RNA targeting, multiplexed with crRNAs targeting conservative sequences of the coronaviruses, has been shown to effectively degrade more than 90% of coronaviruses, including SARS-CoV-2 sequences and live influenza A virus (IAV) in human lung epithelial cells [226].

Finally, guided transcriptional regulators generated by incorporating a repressor or activator domain to the inactivated Cas9 protein, named CRISPR interference (CRISPRi) and CRISPR activation (CRISPRa) respectively, have also been investigated for disease treatment. Thakore et al. used intein-split dual AAV8 vectors to deliver dCas9-KRAB into the mouse liver to target *Pcsk9*, which successfully repressed *Pcsk9* expression and lowered the cholesterol levels in mice with LPL deficiency [153]. Colasante et al. reported that guided transcription activation using AAV-delivered dCas9-VP64 upregulated *Scn1a* gene expression to attenuate seizures in a *Scn1a* haploinsufficiency mouse model of Dravet syndrome [154]. Besides the examples mentioned above, additional gene editing strategies and preclinical studies could also be found in recent reviews [28, 227, 228]. Altogether, these new CRISPR tools showed potentials to treat human diseases through distinct strategies, which warrant more investigations to improve delivery efficiency and address safety concerns in the future.

## Conclusions

In this review, we have provided an overview of milestones achieved, current trends, and challenges of gene therapy using AAV vectors. Gene therapy is a multidisciplinary field, significant innovations have been made in the areas of gene editing, vector engineering, nanoparticles, and other technology platforms. The clinical application of using AAV vector as a tool for gene delivery already has a long history of success in preclinical and clinical studies. Currently, the AAV vector is the leading platform for in vivo gene therapy delivery. As with other viral vectors, the toxicity associated with high-dose AAV delivery and risk for inadvertent insertional mutagenesis are the major concerns and warrant further investigation in clinical applications. With the advancement of CRISPR/Cas9 genome editing technology, AAV vector carrying the CRISPR components have been an attractive tool, with therapeutic potentials validated in both in vivo and ex vivo gene editing. Given the encouraging results and continuous expansion of the CRISPR toolbox, the AAV-CRISPR approach will expand the repertoire of gene therapy strategies and pave the way to the new era of innovative medicine. Further improvement in the performance of engineered AAV capsids and mass production technology is essential to streamline the development of any type of AAV-based therapeutics to deliver the cures for diseases, while further confirmation of the safety in using CRISPR in vivo is needed to harness the full potential of the AAV-CRISPR system for gene editing therapy.

## Data Availability

This article reviews literature and therefore does not contain any associated data and materials.
